# Genotype-dependent changes of gene expression during somatic embryogenesis in oil palm hybrids (*Elaeis oleifera* x *E*. *guineensis*)

**DOI:** 10.1371/journal.pone.0209445

**Published:** 2018-12-31

**Authors:** Ivonaldo Reis Santos, Mariana Rocha Maximiano, Raphael Ferreira Almeida, Raimundo Nonato Vieira da Cunha, Ricardo Lopes, Jonny Everson Scherwinski-Pereira, Angela Mehta

**Affiliations:** 1 Embrapa Recursos Genéticos e Biotecnologia, Brasília, DF, Brazil; 2 Programa de Pós-Graduação em Botânica, Universidade de Brasília, Brasília—DF, Brazil; 3 Programa de Pós-Graduação em Ciências Biológicas (Imunologia e DIP/Genética e Biotecnologia), Universidade Federal de Juiz de Fora, Juiz de Fora, MG, Brazil; 4 Embrapa Amazônia Ocidental, Manaus, AM, Brazil; University of Naples Federico II, ITALY

## Abstract

To understand the molecular processes triggered during the different steps of somatic embryogenesis (SE) in oil palm, the expression of 19 genes associated to SE identified in proteomic and transcriptomic studies was investigated by qRT-PCR. To evaluate the differential expression of these genes, two interspecific hybrid genotypes (*Elaeis oleifera* x *Elaeis guineensis*) contrasting for the acquisition of embryogenic competence were used. Aclorophyllated leaves of both hybrids, one responsive (B351733) and the other non-responsive (B352933) to SE were submitted to callus induction and collected at different time points: 0 (before induction), 14, 30, 90 and 150 days of callus induction (doi). The results obtained showed that all evaluated genes were downregulated at 14 doi in the responsive genotype when compared to the non-responsive. It was also possible to observe that most of the genes changed their expression behavior at 30 doi and were upregulated thereafter until 150 doi, with the exception of the pathogenesis-related PRB1-3-like (*PRB1-3*) gene, which did not show differential expression at 30 doi and was downregulated at 90 and 150 doi when compared to the non-responsive hybrid. These results indicate that 30 doi is a turning point in gene expression, probably associated to embryogenic competence acquisition. We also show that the expression behavior of the responsive genotype is more stable than that of the non-responsive when the different induction time points are compared to 0 doi (before induction). Moreover, the results obtained in this study corroborate our hypothesis that the regulation of genes involved in the control of oxidative stress and energy metabolism are crucial for the acquisition of embryogenic competence in oil palm.

## Introduction

Oil palm is an important crop in the tropics [1] that produces two oils of great economic importance, commonly known as palm oil and palm kernel oil, obtained from the mesocarp and endosperm, respectively [2]. The palm oil is mainly used for cooking, preparing margarine and also for non-food applications (fuel, soap, detergent, cosmetics, etc) [3]. The increasing demand for palm oil is due to the rapid population growth and economic development of several countries [4].

The American palm (*Elaeis oleifera*), known as caiaue or American oil palm, is found in humid tropical America, widely dispersed from Central to Southern America [5–7]. *E*. *oleifera* is considered a promising genetic resource for oil palm breeding programs, since it represents an important source of genetic variability. Among the beneficial agronomic traits are reduced trunk growth rate, reduced size and lower operating costs, resistance to fatal yellowing, as well as high content of unsaturated fatty acids, which gives greater fluidity to the oil under natural conditions, allowing its exploitation for biodiesel production [8–10]. The African oil palm (*E*. *guineensis* Jacq.) is originated from West Africa [11] and is also known as dendem, coconut-palm and palm of guinea. In the last years, due to the presence of diseases, plantations of interspecific hybrids, obtained from the cross between *E*. *oleifera* and *E*. *guineensis* (American and African oil palm, respectively), have increased substantially in Latin America due to their apparent partial resistance to the bud rot disease caused by *Phytophthora palmivora* [12]. *E*. *guineensis* Jacq and *E*. *oleifera* are two monocotyledonous species with a single apical meristem that does not present tillering, and therefore these species cannot be propagated vegetatively by conventional techniques. Therefore, in these species, micropropagation through somatic embryogenesis (SE) is one of the only alternatives for clonal propagation [13].

SE is the process by which cells differentiate to form embryos, reorganizing their epigenetic properties and cell cycle for tissue formation in morphological stages similar to those obtained in zygotic embryogenesis (ZE) [14]. Since the first reports of SE in *E*. *guineensis* by Staritsky [15] and Rabechault et al [16], several studies were performed focused on the induction, proliferation, regeneration and protein profiling in the different SE steps in oil palm [13, 17–19]. The optimization of the protocols has been the central aim of the research, however studies on specific stages of the process such as multiplication, maturation and conversion of somatic embryos remain limited in many palm species [14].

Although millions of plants of different species are produced annually through SE, the molecular mechanisms governing the different steps of the process are not well understood [20]. Thus, the key issue in SE is to understand what triggers cells to change their fate and become embryogenic. Proteomic and gene expression studies, including transcriptomic analyses, during somatic embryogenesis in oil palm were performed and several genes potentially involved in SE were identified [21–25]. Genes have also been described as specific markers of SE in plants, such as the well known *SERK* gene (Somatic Embryogenesis Receptor Kinase), first described in competent carrot cell cultures (*DcSERK*) [26]. SERK has been used as an SE marker to differentiate competent from non-competent cells [27]. Other examples of genes activated and differentially expressed during SE are those encoding glutathione S-transferases (GSTs), which is characteristic of the transition to the development of somatic embryos in *in vitro* culture systems [28], as well as a number of embryogenesis related genes, such as Late-Embryogenic Abundant (*LEA*), Baby Boom (*BBM*), Agamous-like 15 (*AGL15*), Leafy Cotyledon 2 (*LEC2*), Leafy Cotyledon 1 (*LEC1*), and Fusca3 (*FUS3*), expressed during both zygotic and somatic embryogenesis [29].

Although these genes have been identified as related to SE, a more detailed analysis of their expression patterns is necessary and could certainly help advance our knowledge on the crucial biological processes that trigger embryogenic competence acquisition. In this context, the objective of this work was to evaluate the expression of genes involved in the acquisition of embryogenic competence by qRT-PCR, in oil palm genotypes contrasting for the acquisition of embryogenic competence. Genes previously identified by our group were selected, as well genes described in the literature with possible involvement in SE. The results obtained in this study contribute to a better understanding of the genes involved in embryogenic competence acquisition and can help optimize the protocols by altering environmental factors that in turn modulate gene expression during the different steps of SE.

## Materials and methods

### Plant material

In this study, two interspecific oil palm F1 hybrids obtained from the cross between *E*. *oleifera* and *E*. *guineensis* Jacq. were used. These hybrids are unique individuals from the Active germplasm Bank and were selected for cloning propagation since they represent the most productive plants among 573 other plants analyzed for fruit production during 4 consecutive years [30]. However, studies from our group showed that one of them was responsive (B351733) to the SE process while the other (B352933) was not (Scherwinski-Pereira, personal communication). In order to study the differential expression of genes potentially involved in the SE process, a relative expression analysis comparing the responsive (R) and the non-responsive (NR) hybrids at 0, 14, 30, 90 and 150 days of callus induction (doi) was performed. Subsequently, a relative expression analysis of the genes throughout the steps (Time-course) for the R and NR genotypes, using 0 days of induction (doi) as the normalize, was also performed.

### Induction of embryogenic callus and sampling

Aclorophyllated leaves of both oil palm hybrids (B351733 and B352933) were collected and submitted to disinfestation with 70% ethanol for 5 min and with 2.5% sodium hypochlorite for 30 min. The leaves were washed with sterile distilled water and then subjected to the callus induction process. For each replicate, 3 Petri dishes were used (10 x 90 mm) containing six explants, measuring about 1 cm^2^. Each plate contained 25 mL of MS culture medium [31], supplemented with 30 g Lˉ^1^ sucrose; 0,5 g Lˉ^1^ glutamine; 0,5 g Lˉ^1^ of hydrolyzed casein; 2,5 g Lˉ^1^ of activated charcoal; 2,5 g Lˉ^1^ Phytagel and 450 μM picloram, as described by Balzon et al [32], with modifications. During the callus induction period, the explants were stored in a growth room, at a temperature of 25 ± 2°C, in the dark. The collection of the plant material (leaf explants) for the analyses was carried out at 0 (before induction), 14, 30, 90 and 150 days of callus induction (doi), macerated in liquid nitrogen and stored at -80°C until further use for RNA extraction. Three biological replicates were analyzed for each hybrid at each time point, totalizing 30 samples.

### RNA Purification and cDNA synthesis

Total RNA was extracted from 0.1 g of tissue using the Trizol method according to Simms et al [33], with modifications. The quantification was performed using a NanoDrop spectrophotometer (ND-1000 UV-Vis -Termo Fisher). The integrity of the isolated RNA was confirmed on agarose gel (1% agarose; TAE Buffer 1X), pre-stained with ethidium bromide (0.5 mg / mL) [34]. Before cDNA synthesis, RNA was treated with Turbo DNAse (Applied Biosystems/Ambion) to eliminate possible contaminations with genomic DNA. The cDNA was synthesized using 2 μg of RNA treated using the Next Generation MMLV RNAse H Minus First-Strand cDNA Synthesis (DNA express). The cDNA was stored at -20°C and used for qRT-PCR.

### Gene selection, primer design and gene ontology (GO) analysis

Proteins potentially involved in the acquisition of embryogenic competence identified in proteomic studies performed by our research group [35] were selected and the corresponding gene was searched for primer design, including *PGM*, *ACT1*, *ANN1*, *CAT2*, *OASA*, *ENO1*, *MDAR5*, *PFP-BETA*, *RUBA*, *PDIL1-4*, *PRB1*, *PRB1-3*, *EF1* and *HSP81-1* (Table 1). Some genes were also selected from previous published studies, such as *LEA*, *FIE2* and *GBSS1* [36], *BGLUC* and *SERk1*[23] as well as the reference genes *PD00380* [37] and *ACT2* [38] (Table 1). The software Primer3 [39] was used to design all the primers, and the absence of amplification and nonspecific products were evaluated using the software OligoAnalyzer 3.1 [40].

**Table 1:** General information on the genes selected for gene expression analysis.

Gene	Accession # GenBank	Gene name	Forward Primer (5’ to 3’)	TM°C	Reverse Primer (5’ to 3’)	TM°C	Amplicon	Primer Efficiency (%)
***PGM***	XM_010924524.2	2,3-bisphosphoglycerate-independent phosphoglycerate mutase	TGGACGCAATAGAGCAAGTG	60.0	GTCCCCCTTTTTATCGAGGA	60.3	116	86
***ACT1***	NM_001319906.1	Actin-3-like	CACTTCCTCATGCCATCCTT	60.1	CTAACAATTTCCCGCTCTGC	59.8	121	84
***ANN1***	XM_010941329.2	Annexin D1	GTCATAGCCACTCGTGCTGA	60.0	CTCCAGAAGTGTCGCCCTTA	60.4	106	82
***CAT2***	NM_001319913.1	Catalase isozyme 2	ATTGGGATCTCCTGGGAAAC	60.1	ACTCCTGGATGTGGGACTTG	60.0	111	86
***OASA***	XM_010919421.2	Cysteine synthase	AATATCATCTGGGGCTGCTG	60.1	GCTCACCAAAGCTAGGGAAA	59.5	101	78
***ENO1***	XM_010909626.2	Enolase-like	GTCAGCGAGTACCCCATTGT	60.0	TCGTCTCCAACAATCTGCAC	59.8	113	87
***MDAR5***	XM_010942780.2	Monodehydroascorbate reductase 5, mitochondrial	AGCCAAGAAGGTTGCCATTA	59.7	GTGCTCCTCGGGAAATATGA	60.0	106	84
***PFP-BETA***	XM_010915114.2	Pyrophosphate—fructose 6-phosphate 1-phosphotransferase subunit beta	TGTGCTCCTGTTGAGGAATG	59.8	CCTTCTTTATCACGGGCTTG	59.9	100	83
***RUBA***	XM_010920378.1	Rubisco large subunit-binding protein subunit alpha	GCGTGGCAGTTATCAAGGTT	60.1	TCCTCTATGGCTGCGAAAGT	60.0	103	85
***HSP81-1***	XM_010914921.2	Heat shock protein 81–1	TTCGGTGTGGGGTTCTACTC	60.0	ATCCCTAGTCACGGTGAACG	60.0	126	87
***PDIL1-4***	XM_010932368.2	Protein disulfide-isomerase	AACAAGCACCCCTTGTCATC	60.0	CAAGCTGCCATCCAGGTAAT	60.1	113	85
***PRB1***	XM_010940037.2	Pathogenesis-related protein 1-like	CCTCGACCCAGTTCAAGTTC	59.7	TTTGCCTTGGCTACCTCATC	60.2	117	87
***PRB1-3***	XM_010943665.2	Pathogenesis-related protein PRB1-3-like	ACTACGCCAACCAGCGAAT	60.7	TCACTCACCCACGAGTTCAC	59.7	127	85
***EF1***	JN003517.1	Elongation factor 1 (EF1)	AGGCTGACTGTGCTGTCCTT	60.1	TCTGCTTCACACCAAGGGTA	59.3	120	87
***LEA***	XP_010927880.1	Putative late embryogenesis abundant (LEA) protein ^a^	TGGGGTTTGTAATCAGCACA	54.5	TGAAGAAAAGGGAGGCTTCA	53.9	176	90
***FIE2***	XP_010906361.1	Polycomb group protein FIE2 isoform X2 ^a^	TAGCCGCACCATAACATTGA	54.3	TTGATGGTCGCTTGTTGGTA	54.4	176	83
***BGLUC***	XP_010938396.1	Beta-glucosidase 22-like ^a^	TCAGTTTGTTCCAACCCACA	54.6	AGTGTGCTTCCCATGAAACC	55.6		84
***SERk1***	XP_010916943.1	Somatic Embryogenesis Receptor Kinase 1 ^a^	GCATCACCTTCCGAGTTAGC	55.8	TCGTATGGATCTGGGGAGAC	55.6	153	87
***GBSS1***	XP_010940833.1	Granule-bound starch synthase 1,chloroplastic/amyloplastic ^a^	AGCCTTGATGCTGCTTTTGT	55.6	GCATCGCACTTCATCTCAAA	53.5	126	88
***PD00380***	EY397675	Predict 40S ribosomal protein S27-2 ^a^^,^ ^b^	GATGGTTCTTCCGAACGATATTGA	63.0	TCACATCCATGAAGAATGAGTTCG	63.0	113	78
***ACT2***	GAJH01031170.1	Actin/mreB/sugarKinase/HSP70 superfamily ^a^^,^ ^b^	CTCAACCCCAAGGCGAAC	80.9	GTAACACCATCTCCCGAGTCAA	80.9	152	81

^a^Genes selected from the literature

^b^Reference genes

Gene ontology (GO) analysis was performed to understand the biological processes in which these genes are involved using Blast2GO software [41]. The genes were grouped into four categories according their GO terms and according to the literature. Although the same gene can be classified in more than one biological process, in this study only one major biological process was assigned to each gene.

### qRT-PCR experiments and data analysis

The qRT-PCR experiments were performed in the thermal cycler 7300 RealTime PCR System (Applied Biosystems). All reactions were composed of 5 μL of Fast SYBR Green Master Mix (Applied Biosystems), 0.2 μL of each primer at an initial concentration of 10 μM (forward and reverse) and 2 μL of single strand cDNA for each sample (diluted 20X). The PCR program used was a step at 95° C for 10 min to activate the Taq polymerase enzyme (hot start), followed by 40 cycles at 95° C for 15 sec, 60° C for 60 seconds. The denaturation curve (melting curve) was analyzed after the end of the amplification to verify the presence of primer dimers and nonspecific products. The program used was 95°C for15 s, 60°C for 60 s, and increase of 0.3°C a up to 95°C. All experiments were performed using three independent biological replicates and for each biological replicate, three technical replicates were analyzed.

The qRT-PCR reactions were performed in 96 well plates and in each plate a negative control without cDNA was placed to confirm the absence of contamination. The raw fluorescence data from all runs were imported into the Real-time PCR Miner software [42], to determine the Cq value and the PCR efficiency. The analyses of expression and statistics were performed using the REST software [43]. The normalization was performed using internal reference genes. To compare the differences in expression between groups, the t-test was used with 4 degrees of freedom, n = 9 (3 biological replicates and 3 techniques for each biological replicate) as determined by the REST software.

## Results and discussion

### Functional classification

To understand the biological processes in which the 19 genes selected for analysis are involved, a gene ontology (GO) search was performed and revealed important roles related especially to stress responses, defense responses, energy metabolism and development (Table 2). These genes appear to participate in the control of stress and adaptation of the genotypes to the *in vitro* condition during SE. Similar results were obtained by Tan et al [18] in proteomic analyses from mature leaves of oil palm with high or low proliferation rate. Genes related to defense responses and development were also reported by Lin et al [22].

**Table 2:** Gene ontology classification (biological process) of the genes potentially involved in embryogenic competence acquisition in oil palm.

Biological process	Accession # GenBank	Description	Gene	GO (Blast2GO)
**Response to stress**	NM_001319913.1	Catalase isozyme 2	*CAT2*	GO:0098869 GO:0055114 GO:0042542
	XM_010942780.2	Monodehydroascorbate reductase mitochondrial isoform X1	*MDAR5*	GO:0055114
	NM_001319906.1	Actin-101-like isoform X1	*ACT1*	-
	XM_010932368.2	Disulfide-isomerase-like	*PDIL1-4*	GO:0045454 GO:0034976
	DW248206.1	Desiccation-related At2g46140	*LEA*	GO:0009269 GO:0009735
**Energy metabolism**	XM_010924524.2	2,3-bisphosphoglycerate-independent phosphoglycerate mutase	*PGM*	GO:0006007
	XM_010909626.2	Enolase 2	*ENO1*	GO:0006096
	XM_010915114.2	Pyrophosphate—fructose 6-phosphate 1-phosphotransferase subunit beta	*PFP-BETA*	GO:0006002 GO:0061615 GO:0015979 GO:0046835
	EY413718.1	Beta-glucosidase 22-like	*BGLUC*	GO:0005975
	XM_010920378.1	Ru large subunit-binding subunit alpha	*RUBA*	GO:0042026 GO:0009658
	DW248696.1	Granule-bound starch synthase chloroplastic amyloplastic	*GBSS1*	GO:0019252
**Development**	XM_010941329.2	Annexin D1-like	*ANN1*	-
	JN003517.1	Elongation factor 1-alpha-like	*EF1*	GO:0006414
	XM_010918641.1	Somatic embryogenesis receptor kinase 2-like	*SERK*	GO:0007165 GO:0006468
	XM_010919421.2	Cysteine synthase	*OASA*	GO:0006535
	DW247791.1	Polycomb group FIE2	*FIE2*	GO:0006349 GO:2000014
**Defense response**	XM_010940037.2	Pathogenesis-related 1-like	*PRB1*	GO:0009607 GO:0006952
	XM_010943665.2	Pathogenesis-related PRB1-3-like	*PRB1-3*	GO:0009607 GO:0006952
	XM_010914921.2	Heat shock protein 81–1	*HSP801-1*	-

### Gene expression analysis by qRT-PCR

In order to evaluate the expression profile of genes potentially involved in the acquisition of embryogenic competence, the relative expression of 19 genes was analyzed comparing the R and NR genotypes at different SE induction steps. We considered 0, 14 and 30 days of induction as initial steps, 90 days of induction as intermediate step and 150 days of induction as later step before the callus formation.

**Fig 1:**
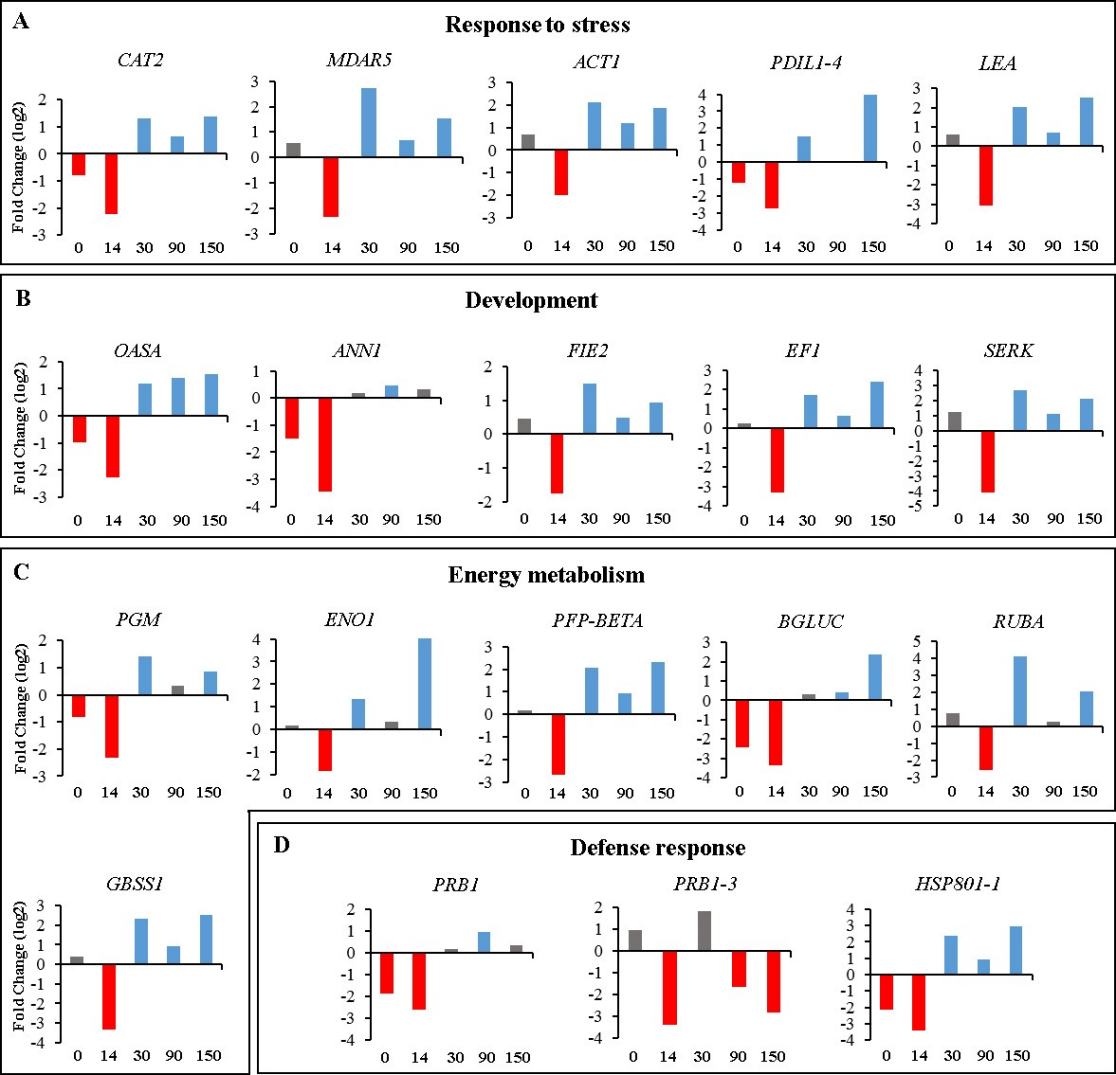
Relative expression analysis of the responsive (R) genotype compared to the non-responsive (NR) genotype at 0, 14, 30, 90 and 150 days of callus induction (doi). **A**- Response to stress; **B**- Development; **C**- Energetic metabolism and **D**- Defense response. The red and blue bars indicate statistically significant (p≤ 0.05) up or downregulated genes, respectively. Gray bars indicate genes that did not show statistical significance.

The results showed that most of the genes analyzed in this study were downregulated at 14 doi when the R genotype was compared with the NR genotype (Fig 1). It was interesting to observe that the relative expression of the majority of the genes analyzed in this comparison changed their expression profile at 30 doi and were upregulated until 150 doi. This upregulation at 30 doi indicates that at this time point the mechanisms of response to the acquisition of embryogenic competence were possibly activated in the R genotype. This increase can be explained by the intense cell division and stretching that occurs at the initiation of callus induction, with the need for synthesis of specific proteins that are responsible for morphological and biochemical alterations [44].

**Fig 2:**
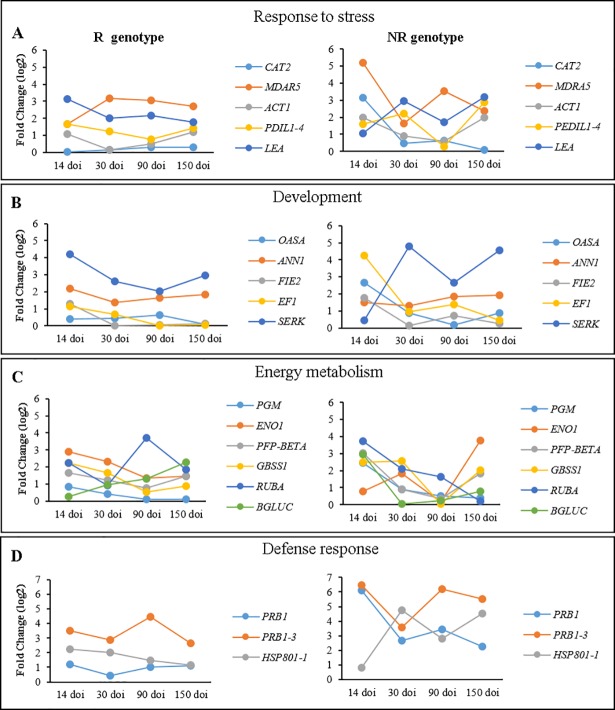
Relative expression analysis of genes throughout the steps (Time-course) for the responsive (R) and non-responsive (NR) genotypes, using 0 days of induction (doi) as the normalizer. **A**- Response to stress; **B**- Development; **C**- Energetic metabolism and **D**- Defense response.

A time-course analysis of the relative expression was also performed for each genotype using the sampling point 0 doi (before induction) as the normalizer. The results showed that the relative expression of the genes throughout the steps in the R genotype is more stable than in the NR genotype (Fig 2). These results suggest that stress levels in the NR genotype are possibly higher in relation to the R genotype, indicating that stress modulation may be crucial for genotype adaptation in the SE induction medium. Indeed, stress adaptation has been reported to influence somatic embryo induction in several plants and promote dedifferentiation [45, 46].

The results obtained indicate that the contrasting responses of acquisition of embryogenic competence between the R genotype and the NR genotype may be related to the increased expression of genes involved in response to stress, defense response, energy metabolism and development, since, at 30, 90 and 150 doi, the expression of the genes involved in these processes had a significant increase in the R genotype when compared to NR genotype. These results indicate that these genes may play an important role in the higher adaptation ability of the R genotype.

### Role of stress response genes during somatic embryogenesis

The first days of callus induction during SE are characterized by the modulation of many genes related to stress, which lead to the assumption that acquisition of SE is an adaptation response of cells cultivated *in vitro* [46]. The control of oxidative stress at adequate levels has been reported as one of the main events for the development SE [47]. The ability to control oxidative stress and preserve protein structure seems to be the key to the success of embryogenic competence acquisition [19]. As part of the response to cellular oxidative stress is the regulation of the expression of genes encoding antioxidant enzymes, which alleviate the damage caused by reactive oxygen species (ROS) [48]. According to Libik et al [49] and Konieczny et al. [50], the increase in the activity of antioxidant enzymes is related to oxidative stress and this stress contributes to accelerate the process of embryogenic response in somatic tissues.

In this study, 6 of the 19 genes evaluated were associated with the response to oxidative stress: *CAT2*, *MDAR5*, *ACT1*, *PDIL1-4* and *LEA* (Table 2). These genes were downregulated at 14 doi and upregulated at 30, 90 and 150 doi in the R genotype when compared to NR (Fig 1A). These results indicate that stress control at 30 doi in the R genotype may be a determining factor for the acquisition of embryogenic competence in oil palm. Therefore, 30 doi seems to be the key moment in which the R genotype adapts to the stress conditions during SE. On the other hand, the decreased expression of the *CAT2*, *MDAR5*, *ACT1*, *LEA* and *PDIL1-4* genes in the NR genotype at 30, 90 and 150 doi may have contributed to the high levels of oxidation that may be one of the causes for the absence of callus formation in this genotype. According to Araldi et al. [51] and Zhou et al. [52], catalase is an antioxidant protein that acts on the regulation of ROS, and its reduced activity results in the accumulation of hydrogen peroxide (H_2_O_2_). The activity of catalase in scavenging H_2_O_2_ was associated with the embryogenic potential of the calli of *Mesembryanthemum crystallinum* [49] and *Larix leptolepis* [53] during SE. Similarly, it was reported in *Lycium barbarum* culture that in the first days of cell differentiation, catalase activity was decreased and its activity was increased with the division of embryogenic cells [54]. Actin has been associated with the process of programmed cell death of the plant cell [55] and, because of this, is considered an effective signaling marker during this process [56]. MDAR5 also has an antioxidant activity [53, 57]; in *Crocus sativus*, *MDAR5* had a high activity from the nodular embryo to the mature embryo [58], proving to be of paramount importance in the SE process.

The *LEA* gene is also related to stress response [59], and was upregulated at 30, 90 and 150 doi in the R genotype when compared to NR. This high expression of the *LEA* gene at these stages is possibly due to the osmotic stress that occurs during the SE induction process. The products of this family of genes may be related to an osmoprotection function, such as defense of cellular structures in mature seed embryos during desiccation and prevention of early germination of zygotic embryos during seed development [60, 61].

In addition to genes encoding antioxidant enzymes, other genes also related to stress in SE, such as defense response genes, were analyzed, including *PRB1*, *PRB1-3* and *HSP801-1* (Table 2). These genes were downregulated at 14 doi when the R genotype was compared to NR (Fig 1D). PR proteins are a heterogeneous group of proteins encoded by genes expressed in response to pathogen attack, but also seem to present related roles in the response to abiotic stresses [62]. Therefore, it is possible that the activation of PRs is related to the adaptation of stress in the induction phase of SE in oil palm.

### Energy metabolism

Energy metabolism proteins have been reported in other SE studies in *E*. *guineensis* showing that some cellular activities under *in vitro* conditions require high energy levels, which explains the relative abundance of these proteins during some stages of SE development [19, 63]. Many studies have been carried out showing the comparison between somatic embryos and zygotic embryos and it has been described that the energy metabolism is more active in somatic embryos [64, 65]. According to Rolland et al. [66], energy metabolism involves the biosynthesis of carbohydrates and lipids, which store energy and are then broken down and consumed in the form of ATP, providing energy for most cellular activities.

In the present study, some genes were related to energy metabolism such as *ENO1*, *PGM*, *PFP-BETA*, *BGLUC*, *RUBA* and *GBSS1* (Fig 1C and Table 2). These genes were downregulated at 14 doi and upregulated at 30, 90 and 150 doi in the R genotype when compared to the NR genotype. The behavior of these genes may indicate a high energy expenditure in callus induction. Thus, high energy consumption requires a high production of ATP, and this may explain the increased expression of these genes at 30, 90 and 150 doi in the R genotype indicating a strong connection to the process of acquisition of embryogenic competence.

Lippert et al [67], also described enolase as involved in the SE process, presenting increased expression. According to Barkla et al [68], enolase has as main role in the conversion of 2-phosphoglycerate to phosphoenolpyruvate (PEP) in the glycolytic pathway, converting glucose into pyruvate, resulting in the final product NADH and ATP. Therefore, a high abundance of enolase is expected [69].

It is important to highlight the participation of the *RUBA* gene in SE. This gene was upregulated at 30, 90 and 150 doi in the R genotype when compared to the NR genotype. This gene has already been reported by Tan et al [18], also showing high abundance in young leaves. It is possible that this gene is related to the photorespiration of the tissue, which prevents the accumulation of toxic products such as 2-phosphoglycerate [70]. Thus, these results may also explain the low oxidative level of the tissues in the R genotype when compared to NR genotype, since *RUBA* could be acting in the control of oxidative stress.

The *PFP-BETA* and *PGM* genes have already been associated with the energy supply during callus induction in *Nelumbo nucifera* Gaertn. spp. *baijianlian*, in the formation and development of *Coffea arabica* torpedo embryos and also in *E*. *guineensis* cell suspensions in response to different concentrations of auxin [71–73]. The *GBSS1* gene is involved in amylose synthesis [74] and histological studies performed by Kanchanapoom and Domyoas [75] and Almeida et al. [35] showed the accumulation of starch granules during the induction of SE oil palm. These results may explain the increased transcription of *GBSS1* at 30, 90 and 150 doi in the R genotype when compared to the NR genotype.

### Genes associated with development

During SE, biochemical and morphological changes occur throughout the development of the induced tissues, which is closely related to changes in gene expression. Several genes are differentially expressed during induction of somatic embryogenesis, while others are expressed during differentiation and maturation of the embryo for plant development [76]. Among the genes associated with development analyzed in this study are *ANN1*, *EF1*, *SERK*, *OASA* and *FIE2* (Table 2). The *ANN1*, *EF1*, *SERK* and *FIE2* genes were downregulated at 14 doi and upregulated at 30, 90 and 150 doi when the R genotype was compared to NR (Fig 1B). It has been described that annexins are related to processes of plant development, such as pollen germination and cotton fiber elongation [77, 78]. Moreover, this gene family has been reported to act in the early steps of somatic embryogenesis from zygotic oil palm embryos, during the somatic embryogenesis of *Coffea arabica* L. and as a molecular marker of the stages of development of somatic embryos of *Manihot esculenta* Crantz [19, 73, 79].

Among the genes involved in the induction of SE, the *SERK* gene plays an important role. This gene was first isolated from embryogenic carrot cells and is considered as a molecular marker for SE [80, 81]. SERK is a leucine rich repeat (LRR) transmembrane protein kinase that increased the capacity of the apical meristem in Arabidopsis to form somatic embryos [82]. The expression of *SERK* is observed in competent cells until the globular stage of the somatic embryo, however, it is not detected in non-embryogenic stages. The expression of this gene was associated with SE in several species, including *Dactylis glomerata* [81], *Arabidopsis thaliana* [82], *Medicago truncatula* [63] and *Helianthus annuus* [83]. Interestingly, in our study, the *SERK* gene showed expression at all time points analyzed (0–150 doi). However, a downregulation was observed at 14 doi followed by an increase from 30–150 doi in the R genotype. These results reinforce our hypothesis that 30 doi is the crucial time point when SE competence is acquired.

Another gene evaluated in our study and related to protein processing was *OASA* (Table 2). This gene was downregulated at 0 doi and at 14 doi and upregulated at 30, 90 and 150 doi in the R genotype when compared to NR. It has already been described in some studies that in the presence of auxin, *OASA* also acts in the regulation of oxidative stress, being able to stimulate cell differentiation and promote the formation of somatic embryos [84, 85]. Thus, this may explain the increase of this gene in the R genotype indicating that this gene could be a potential marker of the acquisition of embryogenic competence.

## Conclusion

The genes evaluated in this study have important roles related mainly to stress and defense response, energy metabolism and development, participating in the control of oxidation and adaptation of the genotypes to *in vitro* conditions during SE induction. The results showed that overall the evaluated genes were downregulated at 14 doi and upregulated from 30 doi to 150 doi when the R genotype was compared with the NR genotype. The results presented here suggest that 30 doi is the key moment when the R genotype changes gene expression leading to the development of somatic embryogenesis. In addition, all genes appear to play a role in the acquisition of embryogenic competence and can be explored as potential markers of SE in oil palm, even PRB1-3 gene that showed a different expression profile, with no modulation at 30 doi and downregulation at 90 and 150 doi in the responsive genotype. The results obtained in this study have an important impact in advancing the current knowledge regarding gene expression during somatic embryogenesis and particularly in understanding the differential response of genotypes to SE. This knowledge is of paramount importance for clonal propagation of elite oil palm genotypes.
